# Dietary Patterns, Weight Perception and Obesity Status, among 10–12-Year-Old Children; an Epidemiological Study in Greece

**DOI:** 10.3390/children8080626

**Published:** 2021-07-23

**Authors:** Aikaterini Kanellopoulou, Rena I. Kosti, Venetia Notara, George Antonogeorgos, Andrea Paola Rojas-Gil, Ekaterina N. Kornilaki, Areti Lagiou, Mary Yannakoulia, Demosthenes B. Panagiotakos

**Affiliations:** 1 Department of Nutrition and Dietetics, School of Health Science and Education, Harokopio University, 17671 Athens, Greece; katerkane@gmail.com (A.K.); gantonogeorgos@gmail.com (G.A.); myianna@hua.gr (M.Y.); 2 Department of Nutrition and Dietetics, School of Physical Education, Sports and Dietetics, University of Thessaly, 42132 Trikala, Greece; renakosti@uth.gr; 3 Laboratory of Hygiene and Epidemiology, Department of Public and Community Health, School of Public Health, University of West Attica, 12243 Athens, Greece; vnotara@uniwa.gr (V.N.); alagiou@uniwa.gr (A.L.); 4 Department of Nursing, Faculty of Health Sciences, University of Peloponnese, 22100 Tripoli, Greece; apaola71@yahoo.com.mx; 5 Department of Preschool Education, School of Education, University of Crete, 74100 Rethimno, Greece; ekornilaki@edc.uoc.gr; 6 Faculty of Health, University of Canberra, Canberra 2617, Australia

**Keywords:** weight perception, children, dietary patterns, obesity, public health, epidemiological study

## Abstract

Adherence to certain dietary patterns influences obesity status in both children and adults. Weight perception influences dietary habits. The aim of this study was to examine children’s dietary habits and obesity status, in relation to weight perception. One thousand seven hundred Greek students enrolled in this study during 2014–2016. Children’s characteristics were assessed through validated questionnaires, and weight status was classified according to the criteria of the International Obesity Task Force. Dietary patterns were assessed through exploratory factor analysis. Overall, 52.2% of children characterized themselves as normal weight, 34.5% as low weight, and 13.3% as overweight/obese; 52.5% of children were in accordance with their actual weight status, with girls being more likely to overestimate their weight. Children followed three dietary patterns, i.e., starchy and protein foods, unhealthy/high-fat foods, and healthy foods. Children who followed the healthy dietary pattern and had accurate weight perception (in accordance with their actual weight), had lower odds of being overweight/obese (*p* < 0.001). Accurate weight perception in conjunction with healthy dietary habits may play a determinant role in the prevention of obesity. From a public health perspective, early identification of children’s weight misperception along with healthy dietary habit promotion shape a crucial role in childhood obesity confrontation.

## 1. Introduction

Obesity is a “modern world” disease with increasing rates in both children and adults, constituting a major public health problem as it adversely affects health, starting in early childhood [1]. It has been characterized as a multifactorial disease [2,3]. Healthy and balanced eating habits, as well as a variety of lifestyle behaviors, mainly physical activity, play an important role and have proven as effective means to control obesity [4,5]. Regarding dietary habits, the vast majority of previous studies have focused on the associations between obesity and single foods, nutrients, and eating behaviors. However, the current trend in nutritional epidemiology is the dietary patterns’ approach, based on a posteriori- or a priori-defined patterns, that provides a more comprehensive and holistic manner of diet, instead of looking separately at nutrients or/and single foods [6,7,8].

Body weight perceptions refer to what a person believes about his/her weight, leading sometimes to a discrepancy in view and the actual weight. Several factors, such as gender, age, peers, family, media, etc., have been proposed that may influence body weight perception in both children and adults [9,10]. Childhood is a critical period of both physical and mental development, and lifestyle behaviors are cultivated. In the transition from childhood to adolescence, body changes as a result of growth spurts may influence weight perception [11], which may play a key role in their dietary habits [12,13]. Indeed, especially for children and adolescents, weight misperceptions may lead to unhealthy eating choices that could modify their growth, development, and, eventually, health [14,15,16,17,18]. Moreover, misperception of weight status may have detrimental psychological influences [11], underlining that perceived weight status, but not the actual weight, was associated with psychological symptoms in adolescence [19], which in turn could influence individuals’ dietary habits. Longitudinal studies have shown that perception of overweight is associated with greater weight gain in individuals [20,21].

The current evidence for how perceived weight status is associated with certain lifestyle behaviors and, in particular, dietary habits, is mixed, demonstrating inconsistent results [12,17,22,23,24], whereas in Greece there is a gap of knowledge in this age group. In addition, research concluded that an accurate perception of weight is crucial for the successful planning of education and behavior intervention programs in the management of adolescents’ obesity [25]. Thus, the aim of the present study was to investigate the association between children’s adherence to a posteriori-defined dietary patterns and obesity status, in relation to weight perception, in Greece. The research hypothesis was that self-perceived weight status interacts with dietary habits, and this interaction influences the likelihood of obesity.

## 2. Materials and Methods

### 2.1. Design and Setting

This is a cross-sectional, school-based study, taking place in five regions of Greece covering almost 75% of the total population, during the school periods 2014–2015 and 2015–2016.

### 2.2. Bioethics

The study was conducted after the approval from the responsible department of the Greek Ministry (F15/396/72005/C1) and following the Declaration of Helsinki (1989). Before the completion of the questionnaires, parental consent was obtained. For more information, the interested reader is referred to [26].

### 2.3. Sample and Sampling Procedures

Overall, a sample of 1728 children 10–12 years old (933 girls), enrolled in the survey. Eligibility criteria of participation were included for all children studying in the two last grades of primary school while no exclusion criteria were applied. In each school, children participated at a rate of about 95–100%. The procedure of sampling lasted during the school months (September–June) and 32 schools from Attica, 5 from Crete, and 10 from the Peloponnese participated. With the contribution of trained researchers (i.e., health visitors, dietitians, registered nurses, and physicians) brief face-to-face interviews with the participants were conducted and the necessary anthropometric measurements were taken. After the checking questionnaires’ completeness, the final working sample for the analyses was *n* = 1700 children. For more information, the interested reader is referred to [26].

### 2.4. Statistical Power Analysis

The sample was sufficient for the assessment of minimum detectable standardized, two-sided differences of 20% on the prevalence of overweight/obesity, with 82% statistical power at a 5% significance level.

### 2.5. Measurements

The survey’s questionnaire included questions about the demographic characteristics, lifestyle activities, habits, perceptions, and knowledge of the participants on health-related risk factors. The Food Frequency Questionnaire (FFQ) [27] and the Physical Activity and Lifestyle Questionnaire (PALQ) [28] were used for the evaluation of children’s dietary habits, sedentary and physical activities, respectively. For more information, the interested reader is referred to [26].

### 2.6. Children’s Weight Status Categorization

Height and weight (in m and Kg, respectively) were measured from all participants through formal procedures for the calculation of the body mass index (BMI). For the evaluation of children’s weight status, the age- and the gender-specific International Obesity Task Force (IOTF) Body Mass Index cut-off criteria were used, which link BMI cut-off criteria at 18 years in child centiles and are appropriate for use as international reference values [29]. One hundred and forty-four children were found to be underweight, 1090 were of normal weight, 380 were overweight, and 87 were obese. Due to the limited number of child participants in the underweight and obesity categories, we combined under-and normal weight categories as well as the overweight and obese categories, as described elsewhere [26].

### 2.7. Assessment of Dietary Patterns

The foods included in FFQ were categorized into eleven food groups (i.e., dairy, soft drinks and concentrated juices, fish, meat, legumes, starchy, ultra-processed food, vegetables, natural juices, fruits, bread). These groups were then analyzed through exploratory factor analysis to identify nutritional patterns that children may follow (see details below).

### 2.8. Assessment of Weight Perception

The children were also asked to express what they believed in terms of their weight (weight perception), using relevant question as described in the study of Martin et al. [30]. The possible answers were categorized in five classes: extremely overweight, slightly overweight, normal weight, slightly underweight, and extremely underweight. For the purposes of the present analysis, the extremely underweight and slightly underweight classes, as well as the slightly overweight and extremely overweight classes, were combined. The same question was also addressed to the parents.

### 2.9. Statistical Analysis

We used mean values and standard deviations to present continuous variables and absolute and relative (%) frequencies to present categorical variables. The independent samples t-test was used to assess the association between normally distributed continuous variables and categorical variables (with two categories). Pearson’s chi-squared test was used to evaluate the associations between categorical variables. To reveal dietary patterns, we applied a factor analysis with the principal components method (PCA) to several food variables. From a database of 41 food variables, we constructed 11 food groups and beverages, which were finally used in the analysis. All food variables used in the analysis had continuous distributions representing the mean weekly consumption of the food groups. For the assessment of the suitability of the data for PCA we applied the Kaiser–Meyer–Olkin (KMO) criterion and Bartlett’s test of sphericity. The dietary patterns were named according to scores (loadings) of the food groups that were larger than 0.4. In order to improve the interpretability of the factors but also to get uncorrelated factors, we used the Varimax method of rotation of the axes. Nested logistic regression models were exploited to assess the potential confounding effect of age, gender, and physical activity, on the association between children’s weight status (dependent outcome) and dietary patterns (independent factors). The choice of the confounding factors was based on a literature review. The results from the logistic regression models are presented as odds ratios (OR), along with their corresponding 95% confidence interval (CI). The variance inflation factor (VIF) was implemented to assess collinearity between the independent variables. Two-sided hypothesis tests were considered. Cohen’s kappa coefficient was used to measure agreement between children’s perception and their actual obesity status. The range of values of this index is from −1 to 1; the value 1 indicates a perfect agreement, −1 indicates a perfect disagreement, while 0 indicates a completely random agreement. For all statistical analyses we used STATA 14.0 (M. Psarros et Assoc, Sparta, Greece).

## 3. Results

### 3.1. Prevalence of Overweight and Obesity

The overall prevalence of overweight children was 22.4% and obesity was 5.1%. Gender-specific analysis revealed that 32.4% of boys and 23.3% of girls were categorized as overweight/obese (*p* < 0.001 for gender difference).

In Table 1, children’s characteristics and eating habits by body weight status are presented. There was a significant association of body weight with male gender (*p* < 0.01) and engagement in physical activities (*p* < 0.01). Specifically, boys were more likely to be overweight/obese compared to girls, and both boys and girls engaged in physical activities were less likely to be overweight/obese compared to those followed sedentary behaviors. Regarding children’s eating habits, those who were classified as overweight/obese reported higher consumption of ultra-processed food (*p* = 0.05) and less consumption of natural juices (*p* = 0.05), whereas no other significant differences were observed concerning dietary habits and overweight/obesity status (Table 1).

### 3.2. Weight Status Perception and Its Determinants

The overall self-perception which was in accordance with actual body weight status (agreement) of children was 52.5% (Table 2). Girls compared to boys showed higher accordance regarding their weight perception and actual weight status (54.3% for girls and 50.3% for boys, *p* = 0.04) There was a fair agreement between children’s perception and their actual obesity status (Cohen’s kappa = 0.207 (95% CI 0.180, 0.234), *p* < 0.001). Girls showed a slightly higher agreement (k = 0.210 (95% CI 0.161, 0.259), *p* < 0.001) than boys (k= 0.202 (95% CI 0.153, 0.251), *p* < 0.001). Generally, children had the tendency to underestimate their weight status since 38.9% of children characterized their weight as less than the actual, whereas only 8.6% of children overestimated their weight status.

Parents were more accurate on their perception of their children’s weight status compared to children (67.2% vs. 52.5, *p* < 0.001). A fair agreement between parents’ and children’s perception was observed (k = 0.337 (95% CI 0.284, 0.390), *p* < 0.001).

A sensitivity analysis by excluding children who were particularly close to the cut-off of the overweight and obesity status (±1 kg/m^2^) was also applied (based on 969 children; 550 girls). The new results regarding the correct classification rates presented were comparable with the ones presented in Table 2. Specifically, a fair agreement between children’s perception and their actual obesity status was observed (k = 0.231 (95% CI 0.192, 0.270), *p* < 0.001). Now, girls showed almost the same agreement (k = 0.231 (95% CI 0.172, 0.290), *p* < 0.001) with boys (k = 0.230 (95% CI 0.175, 0.285), *p* < 0.001). Moreover, the overall self-perception was also in accordance with actual body weight status (agreement) of children, although it was a little smaller (49.6% for all children, 48.4% for boys, and 50.7% for girls). There was no significant difference in agreement rates either for all children or by gender (*p* ≥ 0.162).

A strong association was observed between children’s weight perception and actual overweight/obesity status (*p* < 0.001). Among children with obesity, only 47.0% had a concordant view of their body weight, whereas 53% viewed their body weight as “normal weight” or “underweight”. The level of underestimation was even higher among overweight boys and girls, i.e., 68% viewed their body weight as “normal weight”.

After adjusting for age, gender, and physical activity status, overweight/obese children had a 3.82 times (95% CI 3.04, 4.90) higher likelihood of underestimating their actual weight status, as compared to the normal weight. Moreover, older children (11–12 years) were 13% less likely to underestimate their body weight as compared to the younger (10 years; OR: 0.87, 95% CI 0.75, 1.00). No gender interaction was observed between actual weight status and self-perception of body weight (*p* = 0.96). In addition, no interaction was observed between children’s physical activity status, actual weight status, and weight self-perception (*p* = 0.67).

### 3.3. Dietary Pattern Analysis

**Figure 1:**
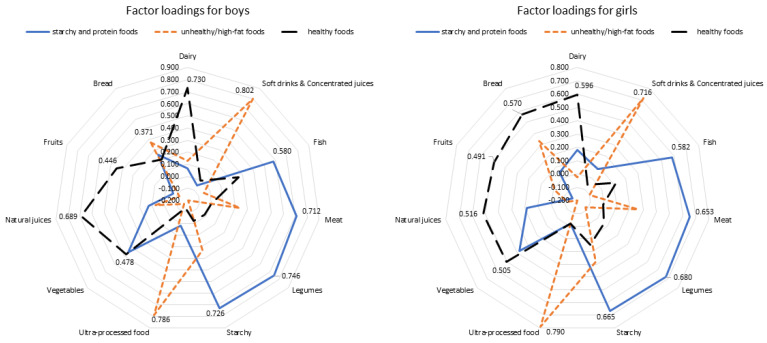
Results from exploratory factor analysis that evaluated children’s dietary habits, split by gender (the extraction method used was the Principal Component Analysis and the rotation method was the varimax with Kaiser normalization).

To further explore children’s dietary habits, a holistic approach followed the single food or food groups analysis. Particularly, a posteriori dietary patterns’ analysis with the application of factor analysis evaluated children’s dietary habits separately in boys and girls. Figure 1 illustrates the results from the factor analysis. In both genders, the analysis revealed three main factors (i.e., dietary components), explaining the 49.4% and 44.2% of the total variance in consumption for boys and girls, respectively. Given that the higher absolute values indicate that the food variable contributes more to the characterization of the component, it could be suggested that the extracted components for boys are characterized as follows: higher consumption of fish, meat, legumes, and starchy foods (Factor 1, named “starchy and protein foods”); higher consumption of soft drinks and concentrated juices, ultra-processed foods, and bread (Factor 2, named “unhealthy/high-fat foods”); higher consumption of dairy, vegetables, natural juices, and fruits (Factor 3, named “healthy foods”. For girls, similar components were extracted (Factors 2 and 3 were extracted in reverse order). Moreover, the higher consumption of bread contributed more to the “healthy foods” pattern. The application of factor analysis to all children led to similar results (the three extracted factors interpreted the 46.8% of the data variability). Thus, in the sequel as a posteriori-defined dietary patterns, we will use the factors revealed from the factor analysis in all children. A total of 37.9% of children followed the “starchy and protein foods” pattern, 38.4% of children followed the “unhealthy/high-fat foods”, while 47.8% of children followed the “healthy foods” pattern. No association was revealed between actual body weight status and adherence to each of the dietary patterns (*p* > 0.365).

After adjusting for age, gender, and physical activity, higher adherence to the “healthy foods” dietary pattern was associated with 0.91 times lower odds of overweight/obesity (95% CI 0.81, 0.98), whereas higher adherence to the “unhealthy/high-fat foods” pattern was associated with 1.11 times (95% CI 1.01, 1.23) increased odds of overweight/obesity. No significant association was observed between “starchy and protein foods” dietary pattern and likelihood of overweight/obesity (1.03, 95% CI 0.93, 1.15).

### 3.4. Dietary Habits, Dietary Patterns, and Self-Perceived Agreement with Actual Weight Status

Afterwards, the analysis was focused on dietary habits and patterns followed by the children according to self-perceived agreement with actual weight status (Table 3). Self-perceived agreement with actual weight status was significantly associated with the consumption of dairy products, meat and products, and legumes. In particular, children who underestimated their weight reported higher consumption of dairy products compared to children with self-perceived weight accuracy (*p* = 0.017), as well those who overestimated their weight (*p* = 0.047). Children with accurate self-perceived weight status had a lower consumption of meat and meat products compared to children who underestimated (*p* = 0.026) as well as those who overestimated their weight (*p* = 0.018). Moreover, children who underestimated or accurately perceived their weight status had a lower consumption of legumes compared to children who overestimated it (*p* = 0.020). Regarding dietary patterns, children who under- or overestimated their actual weight status had higher scores (i.e., closer) on the “Starchy and protein food” dietary pattern as compared to those who accurately perceived their weight (Table 3).

### 3.5. Dietary Patterns and Children’s Overweight/Obesity Status by Self-Perceived Agreement with Actual Weight Status

**Figure 2:**
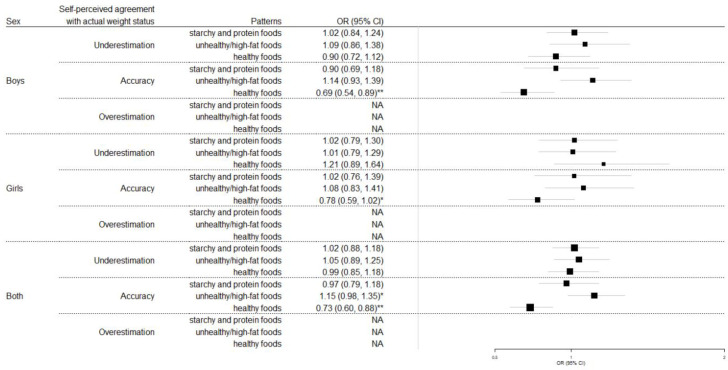
Results from multiple logistic regression models that evaluated the association between children’s dietary patterns and their obesity status, by self-perceived agreement with actual weight status. * *p* < 0.1, ** *p* < 0.05. NA: Logistic regression is not applicable as all children are categorized as normal-weighted, given that children with obesity/overweight cannot overestimate their weight status.

A significant interaction was observed between adherence to dietary patterns and self-perceived agreement with actual weight status on children’s overweight/obesity status (*p* < 0.001). Thus, to explore the second aim of this study, i.e., the association between children’s dietary patterns and obesity status in the light of self-perceived agreement with actual weight status, stratified multiple logistic regression models were applied (Figure 2). Among those who correctly classified themselves in the body weight category, greater adherence to the “unhealthy/high-fat foods” dietary pattern was associated with increased likelihood to be overweight/obese, whereas greater adherence to the “healthy foods” pattern was associated with decreased likelihood of being overweight/obese. However, when the analysis was stratified by gender, the findings regarding the “unhealthy/high-fat foods” dietary pattern lost its significance in both boys and girls; however, the favorable association of the “healthy” dietary pattern with overweight/obesity status was still evident in both boys and girls. No associations were observed regarding dietary patterns and body weight status of children when the analysis was focused on those who underestimated or overestimated their actual body weight status.

## 4. Discussion

In the present study, the association between adherence to a posteriori-defined dietary patterns and the overweight/obesity status of schoolchildren, by self-perceived weight status, was investigated. Almost one out of two children had a misperception of their body weight status, and this was more evident among boys. The “healthy” dietary pattern was associated with a lower likelihood of a child being overweight/obese, and borderline greater adherence to the “unhealthy/high-fat foods” dietary pattern was associated with increased likelihood, but only among children with accurate weight perception, suggesting a moderating effect of children’s weight perception on the diet–obesity relationship. Despite the limitations of the present study due to its observational design, the messages carried are of particular interest from a public health perspective to better understand the diet–obesity relationship in children.

A posteriori dietary pattern analysis considers the cumulative and interactive effects among dietary components to reflect the complexity of the human diet. It is a comprehensive, and “holistic” approach that overcomes several methodological barriers in studying the association between human diet and health. The present study, in line with the findings of the most recently conducted systematic review [31], confirmed that the dominant unhealthy dietary pattern that children follow, and appears to significantly affect their weight status, is the “high-fat foods” pattern, characterized by obesogenic foods, i.e., rich in soft drinks and concentrated juices, ultra-processed foods (such as croissants, chocolate, cookies, chips), and bread. This may be because children’s dietary choices are potentially influenced by the standards displayed through the promotion and advertising of various unhealthy food products [32].

In accordance with other studies [22,30,33,34], in the present study only half of the participated children viewed their body weight status in agreement with actual weight, and gender seemed to play a decisive role. In previous studies [35], the percentage of agreement was higher in girls compared to boys, which could be attributed to the fact that girls are more interested in their appearance from an early age [36]. In the present study, it was revealed that children with borderline values of BMI seem to not affect the correct classification rates. This is at odds with previous findings [37], that adolescents who were on the extreme edges of their weight category were more likely to misperceive their weight status. This prompted the authors to speculate that, beyond differences in study design, in late childhood, weight misperception could imply more “ignorance in accurate perception” rather than “Willful ignorance”, probably as a result of the greater influence from media and cultural norms at a later age stage. In line with the literature, children have a higher tendency to misperceive their weight status compared to the parental misperception of children’s weight status [38].

However, our analysis also revealed that girls were more likely to overestimate their weight, as compared to boys who tended to underestimate their weight, in line with previous studies [14,33,34,39]. The apparent consequences of misperceptions of being overweight in the eating disorder field may lead girls, in the future, to unhealthy weight-control practices [15,40]. However, further concerns have been also expressed regarding misperceptions of underestimation, which may lead to unfavorable health behaviors [34]. In particular, overweight adolescents who underestimate their weight may engage less in health behavior changes due to a lack of motivation, disregarding messages about nutrition and exercise as not relevant to them [33,37,41]. On the other hand, evidence supports that perceiving oneself as overweight was predictive of increased weight gain over time [20,21,42]. The behavioral mechanisms behind this observation could be attributed either to the adoption of unhealthy behaviors due to psychosocial reasons [14,43], perceiving oneself as belonging to a stigmatized group, or through the engagement of health-compromising weight-loss methods [40] leading to disordered eating [15]. Indeed, children’s self-perception of their weight, whether it is accurate or not, may affect their weight fluctuation [44], predicting weight-control intentions, regardless of objective weight status [22,34].

A main goal of the present study was to evaluate children’s weight perception and its potential role in the diet–obesity relationship. In line with the findings of other studies [12,22,23] that accuracy in body weight perception was positively correlated with healthier dietary choices, in the present study it was revealed that children who had weight status misconceptions (i.e., discordance) tended to follow a less healthy dietary pattern characterized as “Starchy and protein food” as compared to those who correctly perceived their weight. It is worth mentioning that the adherence to a ‘‘starchy and protein food” pattern could eventually lead to obesity through several biological mechanisms [45,46,47].

The hypothesis between body weight perception, dietary habits, and children’s obesity status is relatively new in the literature; our study is one of the first which revealed that the protective role of a healthy dietary pattern in obesity status was evident only among children with accurate self-perception of their weight. This finding is extremely important, revealing that the hidden agent behind “accuracy” in self-perception of weight status likely has a solid psychological background. Indeed, weight misperception was a strong predictor of body dissatisfaction [48], which in turn could influence weight status [49]. This speculation could be further justified by the fact that higher self–esteem was associated with higher odds of accurate perception of being overweight in females [50], whereas weight perception of normal weight, regardless if it is accurate or not, implied positive psychological wellbeing associated with dietary habits more favorable for health among adolescents across the weight range [13,51]. Indeed, misperception of weight status in adolescence is associated with distorted body image, and higher scores for general psychopathology [52]. Therefore, in line with the notion that “perception” is more crucial and powerful than “reality” in terms of weight status [53], it seems that adolescents with accurate perception of their weight status adopt a healthy dietary pattern likely because they feel “comfortable about themselves”, leading to lower odds of obesity.

### Limitations

Given that this was an observational study, some limitations should be acknowledged. First, no causal inferences can be made regarding children’s weight perceptions, dietary habits adopted, and overweight/obesity status. The obesity categories used here were based on standard adult BMI calculations that are back-extrapolated to cutoffs proposed by the IOTF’s reference standard, which is a widely accepted approach to standardize obesity cutoffs across the world, despite the argument about adopting it as it may be more appropriate for larger population-based studies. However, our sample was relatively large and representative of a broader area of Greece. Moreover, the evaluation of only 10–12 year-old-children is another limitation, but the aim of the study was to focus on pre-adolescents that are also able to respond themselves. Reporting bias due to the self-reporting questionnaires may also be a potential limitation. However, the validity of the given responses can be ensured by the presence of a trained investigator throughout the completion of the questionnaire.

## 5. Conclusions

The finding that one half of children described their weight status inaccurately and among them, no association was observed between diet–obesity, deserves further attention as it may have serious complications in their future health status. Accurate weight perception in conjunction with healthy dietary habits may play a determinant role in the prevention of obesity. Thus, the early identification of children with wrong weight perception, along with the promotion of healthy dietary habits, is of crucial importance from a public health perspective to combat childhood obesity.

## Figures and Tables

**Table 1:** Children’s socio-demographic characteristics and eating habits, by body weight status.

Children’s Characteristics	Overall (*n* = 1700)	Normal Weight ** (*n* = 1233)	Overweight and Obese ** (*n* = 467)	*p* *
Age	11.21 (0.79)	11.23 (0.79)	11.15 (0.78)	0.058
Gender (boys/girls)				
Boys	781 (45.9%)	528 (42.8%)	253 (54.2%)	<0.001
Girls	919 (54.1%)	705 (57.2%)	214 (45.8%)	
Physical activity (yes)	1243 (73.1%)	923 (80.8%)	320 (73.1%)	<0.001
**Children’s dietary habits** (times/week), mean (SD)				
Dairy	3.76 (1.65)	3.77 (1.65)	3.73 (1.65)	0.623
Soft drinks and Concentrated juices	1.24 (1.56)	1.19 (1.46)	1.36 (1.80)	0.079
Fish	0.67 (0.87)	0.67 (0.84)	0.66 (0.95)	0.862
Meat and products	1.60 (1.27)	1.57 (1.24)	1.68 (1.35)	0.102
Legumes	1.83 (1.68)	1.84 (1.66)	1.82 (1.75)	0.812
Starchy	1.64 (1.09)	1.62 (1.05)	1.67 (1.16)	0.421
Ultra-processed food	1.41 (1.63)	1.36 (1.57)	1.54 (1.77)	0.050
Vegetables	2.06 (1.54)	2.06 (1.50)	2.07 (1.65)	0.839
Natural juices	3.71 (2.69)	3.79 (2.69)	3.50 (2.69)	0.050
Fruits	2.66 (1.69)	2.67 (1.67)	2.63 (1.75)	0.740
Bread	2.17 (1.67)	2.20 (1.69)	2.10 (1.64)	0.325

Children’s eating habits are presented as mean (SD) and categorical variables as frequencies (%), * Level of significance set at *p* < 0.05; tested via independent samples *t*-test for eating habits, and chi-square test for all other categorical variables ** Weight status is defined based on BMI cut-offs for adults and on IOTF cut-off criteria for children. SD = Standard deviation; IOTF = International obesity task force; BMI = Body mass index.

**Table 2:** Results from a classification analysis between actual BMI status and self-perceived body weight of children.

			Actual Body Weight Status (IOTF)			
			Underweight	Normal	Overweight/Obese	% Agreement
All children	Self-perceived body weight	Low weight	**77 (55.4%)**	344 (33.8%)	126 (29.4%)	52.5%
		Normal	61 (43.9%)	**609 (59.9%)**	157 (36.6%)	
		Overweight/Obese	1 (0.7%)	64 (6.3%)	**146 (34.0%)**	
Boys		Low weight	**29 (50.9%)**	160 (36.9%)	69 (30.4%)	50.3%
		Normal	28 (49.1%)	**255 (58.8%)**	81 (35.7%)	
		Overweight/Obese	0 (0.0%)	19 (4.4%)	**77 (33.9%)**	
Girls		Low weight	**48 (58.5%)**	184 (31.6%)	57 (28.2%)	54.3%
		Normal	33 (40.2%)	**354 (60.7%)**	76 (37.6%)	
		Overweight/Obese	1 (1.2%)	45 (7.7%)	**69 (34.2%)**	

IOTF = International obesity task force; BMI = Body mass index. The rates of children’s self-perceptions about their body weight which was in accordance with their actual BMI status are presented in bold. The rates for children’s underestimation are presented in the light grey colored cells. The rates for children’s overestimation are presented in the deep grey colored cells.

**Table 3:** Children’s dietary habits and patterns, by self-perceived agreement with actual weight status.

	Underestimation (*n* = 627)	Accuracy (*n* = 832)	Overestimation (*n* = 126)	*p* *
**Children’s dietary habits** (times/week), mean (SD)				
Dairy	3.93 (1.66) *	3.71 (1.61)	3.60 (1.82)	**0.025**
Soft drinks and Concentrated juices	1.24 (1.44)	1.25 (1.66)	1.14 (1.25)	0.762
Fish	0.74 (0.97)	0.62 (0.81)	0.64 (0.65)	0.116
Meat and products	1.67 (1.32) *	1.52 (1.18)	1.81 (1.45) *	**0.014**
Legumes	1.88 (1.70)	1.75 (1.61)	2.13 (1.97) *	**0.047**
Starchy	1.69 (1.13)	1.60 (1.08)	1.65 (0.89)	0.353
Ultra-processed food	1.38 (1.54)	1.42 (1.67)	1.33 (1.56)	0.776
Vegetables	2.12 (1.62)	1.99 (1.46)	2.10 (1.47)	0.307
Natural juices	3.75 (2.71)	3.70 (2.68)	3.69 (2.73)	0.944
Fruits	2.56 (1.66)	2.70 (1.71)	2.91 (1.73)	0.216
Bread	2.17 (1.69)	2.16 (1.68)	2.14 (1.55)	0.982
**Children’s dietary patterns**, mean factor score (SD)				
Starchy and protein food	0.06 (1.05) *	−0.07 (0.94)	0.12 (0.94) *	**0.013**
Unhealthy/high-fat foods	0.01 (0.04)	0.01 (1.06)	−0.08 (0.81)	0.621
Healthy foods	0.04 (0.98)	−0.01 (1.01)	−0.05 (1.05)	0.483

Children’s eating habits are presented as mean (SD), * *p* < 0.05; associations were tested via Analysis of Variance (ANOVA), while post-hoc analyses via independent samples *t*-test (with “accuracy” as the reference category) and chi-square test for all other categorical variables, after correcting the *p*-values using the Bonferoni rule. The numbers in bold show statistically significant results. SD = Standard deviation.

## Data Availability

Data are available upon request.

## References

[1] Barroso W.K.S., Souza A.L.L. (2020). Obesity, Overweight, Body Adiposity and Cardiovascular Risk in Children and Adolescents. Arq. Bras. Cardiol..

[2] Kyle T.K., Dhurandhar E.J., Allison D.B. (2016). Regarding obesity as a disease: Evolving policies and their implications. Endocrinol. Metab. Clin. North Am..

[3] Bray G., Kim K., Wilding J., Federation W.O. (2017). Obesity: A chronic relapsing progressive disease process. A position statement of the World Obesity Federation. Obes. Rev..

[4] Kremers S.P. (2010). Theory and practice in the study of influences on energy balance-related behaviors. Patient Educ. Couns..

[5] Brug J., Lien N., Klepp K.I., van Lenthe F.J. (2010). Exploring overweight, obesity and their behavioural correlates among children and adolescents: Results from the Health-promotion through Obesity Prevention across Europe project. Public Health Nutr..

[6] Nelson M.E., Hamm M.W., Hu F.B., Abrams S.A., Griffin T.S. (2016). Alignment of healthy dietary patterns and environmental sustainability: A systematic review. Adv. Nutr..

[7] Panagiotakos D.B., Notara V., Kouvari M., Pitsavos C. (2016). The Mediterranean and other dietary patterns in secondary cardiovascular disease prevention: A review. Curr. Vasc. Pharmacol..

[8] Tapsell L.C., Neale E.P., Satija A., Hu F.B. (2016). Foods, nutrients, and dietary patterns: Interconnections and implications for dietary guidelines. Adv. Nutr..

[9] Gregory C.O., Blanck H.M., Gillespie C., Maynard L.M., Serdula M.K. (2008). Health perceptions and demographic characteristics associated with underassessment of body weight. Obesity.

[10] Kim K.H.c. (2007). Religion, weight perception, and weight control behavior. Eat. Behav..

[11] Xie B., Liu C., Chou C.p., Xia J., Spruijt-Metz D., Gong J., Li Y., Wang H., Johnson C.A. (2003). Weight perception and psychological factors in Chinese adolescents. J. Adolesc. Health.

[12] Buscemi S., Marventano S., Castellano S., Nolfo F., Rametta S., Giorgianni G., Matalone M., Marranzano M., Mistretta A. (2018). Role of anthropometric factors, self-perception, and diet on weight misperception among young adolescents: A cross-sectional study. Eat. Weight Disord..

[13] Mbogori T., Arthur T.M. (2021). Perception of Body Weight Status Is Associated with the Health and Food Intake Behaviors of Adolescents in the United States. Am. J. Lifestyle Med..

[14] Khambalia A., Hardy L.L., Bauman A. (2012). Accuracy of weight perception, life-style behaviours and psychological distress among overweight and obese adolescents. J. Paediatr. Child Health.

[15] Brechan I., Kvalem I.L. (2015). Relationship between body dissatisfaction and disordered eating: Mediating role of self-esteem and depression. Eat. Behav..

[16] Fan M., Jin Y. (2015). The effects of weight perception on adolescents’ weight-loss intentions and behaviors: Evidence from the youth risk behavior surveillance survey. Int. J. Environ. Res. Public Health.

[17] Fredrickson J., Kremer P., Swinburn B., de Silva A., McCabe M. (2015). Weight perception in overweight adolescents: Associations with body change intentions, diet and physical activity. J. Health Psychol..

[18] Goldschmidt A.B., Wall M.M., Loth K.A., Neumark-Sztainer D. (2015). Risk factors for disordered eating in overweight adolescents and young adults. J. Pediatric Psychol..

[19] Tang J., Yu Y., Du Y., Ma Y., Zhu H., Liu Z. (2010). Association between actual weight status, perceived weight and depressive, anxious symptoms in Chinese adolescents: A cross-sectional study. BMC Public Health.

[20] Klein E.G., Lytle L.A., Chen V. (2008). Social ecological predictors of the transition to overweight in youth: Results from the Teens Eating for Energy and Nutrition at Schools (TEENS) study. J. Am. Diet. Assoc..

[21] Sutin A.R., Terracciano A. (2015). Body weight misperception in adolescence and incident obesity in young adulthood. Psychol. Sci..

[22] Edwards N.M., Pettingell S., Borowsky I.W. (2010). Where perception meets reality: Self-perception of weight in overweight adolescents. Pediatrics.

[23] Southerland J., Wang L., Richards K., Pack R., Slawson D.L. (2013). On Academics Misperceptions of Overweight: Associations of Weight Misperception with Health-Related Quality of Life among Normal-Weight College Students. Public Health Rep..

[24] Thunfors P., Hanlon A., Collins B. Weight status misperception and the health behaviors of obese adolescents. https://print.ispub.com/api/0/ispub-article/11452.

[25] Gaylis J.B., Levy S.S., Hong M.Y. (2020). Relationships between body weight perception, body mass index, physical activity, and food choices in Southern California male and female adolescents. Int. J. Adolesc. Youth.

[26] Kanellopoulou A., Notara V., Antonogeorgos G., Chrissini M., Rojas-Gil A.P., Kornilaki E.N., Lagiou A., Panagiotakos D.B. (2021). Inverse Association Between Health Literacy and Obesity Among Children in Greece: A School-Based, Cross-Sectional Epidemiological Study. Health Educ. Behav..

[27] Antonogeogros G., Grigoropoulou D., Papadimitriou A., Priftis K., Anthracopoulos M., Nicolaidou P., Panagiotakos D. (2011). Validation of a Food Frequency Questionnaire designed for children 10-12 years: The Panacea-FFQ. Pediatric Res..

[28] Argiropoulou E.C., Michalopoulou M., Aggeloussis N., Avgerinos A. (2004). Validity and reliability of physical activity measures in Greek high school age children. J. Sports Sci. Med..

[29] Cole T.J., Bellizzi M.C., Flegal K.M., Dietz W.H. (2000). Establishing a standard definition for child overweight and obesity worldwide: International survey. BMJ.

[30] Martin M.A., Frisco M.L., May A.L. (2009). Gender and race/ethnic differences in inaccurate weight perceptions among US adolescents. Women’s Health Issues.

[31] Liberali R., Kupek E., Assis M.A.A.d. (2020). Dietary patterns and childhood obesity risk: A systematic review. Child. Obes..

[32] Cairns G., Angus K., Hastings G., Caraher M. (2013). Systematic reviews of the evidence on the nature, extent and effects of food marketing to children. Retrosp. Summary. Appet..

[33] Jackson S.E., Johnson F., Croker H., Wardle J. (2015). Weight perceptions in a population sample of English adolescents: Cause for celebration or concern?. Int. J. Obes..

[34] Patte K.A., Laxer R., Qian W., Leatherdale S.T. (2016). Weight perception and weight-control intention among youth in the COMPASS study. Am. J. Health Behav..

[35] Aloufi A.D., Najman J.M., Al Mamun A. (2019). Predictors of persistent body weight misclassification from adolescence period to adulthood: A longitudinal study. J. Epidemiol. Glob. Health.

[36] Dixey R., Sahota P., Atwal S., Turner A. (2001). “Ha ha, you’ re fat, we’re strong”; A qualitative study of boys’ and girls’ perceptions of fatness, thinness, social pressures and health using focus groups. Health Educ..

[37] Fredrickson J., Kremer P., Swinburn B., de Silva-Sanigorski A., McCabe M. (2013). Biopsychosocial correlates of weight status perception in Australian adolescents. Body Image.

[38] Blanchet R., Kengneson C.C., Bodnaruc A.M., Gunter A., Giroux I. (2019). Factors Influencing Parents’ and Children’s Misperception of Children’s Weight Status: A Systematic Review of Current Research. Curr. Obes. Rep..

[39] Fan M., Jin Y., Khubchandani J. (2014). Overweight misperception among adolescents in the United States. J. Pediatric Nurs..

[40] Stephen E.M., Rose J.S., Kenney L., Rosselli-Navarra F., Weissman R.S. (2014). Prevalence and correlates of unhealthy weight control behaviors: Findings from the national longitudinal study of adolescent health. J. Eat. Disord..

[41] Deschamps V., Salanave B., Chan-Chee C., Vernay M., Castetbon K. (2015). Body-weight perception and related preoccupations in a large national sample of adolescents. Pediatric Obes..

[42] Haynes A., Kersbergen I., Sutin A., Daly M., Robinson E. (2018). A systematic review of the relationship between weight status perceptions and weight loss attempts, strategies, behaviours and outcomes. Obes. Rev..

[43] Roberts R.E., Duong H.T. (2013). Perceived weight, not obesity, increases risk for major depression among adolescents. J. Psychiatr. Res..

[44] Chung A.E., Perrin E.M., Skinner A.C. (2013). Accuracy of child and adolescent weight perceptions and their relationships to dieting and exercise behaviors: A NHANES study. Acad. Pediatrics.

[45] Aller E.E., Abete I., Astrup A., Martinez J.A., Baak M.A.v. (2011). Starches, sugars and obesity. Nutrients.

[46] Koletzko B., Demmelmair H., Grote V., Prell C., Weber M. (2016). High protein intake in young children and increased weight gain and obesity risk. Am. J. Clin. Nutr..

[47] Wilcox G. (2005). Insulin and insulin resistance. Clin. Biochem. Rev..

[48] Knowles G., Ling F.C., Thomas G.N., Adab P., McManus A.M. (2015). Body size dissatisfaction among young Chinese children in Hong Kong: A cross-sectional study. Public Health Nutr..

[49] Pallan M.J., Hiam L.C., Duda J.L., Adab P. (2011). Body image, body dissatisfaction and weight status in South Asian children: A cross-sectional study. BMC Public Health.

[50] Perrin E.M., Boone-Heinonen J., Field A.E., Coyne-Beasley T., Gordon-Larsen P. (2010). Perception of overweight and self-esteem during adolescence. Int. J. Eat. Disord..

[51] Patte K.A., Laxer R.E., Qian W., Leatherdale S.T. (2016). An analysis of weight perception and physical activity and dietary behaviours among youth in the COMPASS study. SSM-Popul. Health.

[52] Jáuregui-Lobera I., Bolaños-Ríos P., Santiago-Fernández M.J., Garrido-Casals O., Sánchez E. (2011). Perception of weight and psychological variables in a sample of Spanish adolescents. Diabetes Metab. Syndr. Obes. Targets Ther..

[53] Robinson E., Hunger J., Daly M. (2015). Perceived weight status and risk of weight gain across life in US and UK adults. Int. J. Obes..

